# Involvement of Nitric Oxide on Calcium Mobilization and Arachidonic Acid Pathway Activation during Platelet Aggregation with different aggregating agonists

**Published:** 2016-03

**Authors:** Debipriya Banerjee, Sahana Mazumder, Asru Kumar Sinha

**Affiliations:** 1 Sinha Institute of Medical Science & Technology, Kolkata, India;; 2 Department of Physiology, Rammohan College, University of Calcutta, Kolkata, India

**Keywords:** acute coronary syndromes, arachidonic acid, calcium mobilization, nitric oxide

## Abstract

Platelet aggregation by different aggregating agonists is essential in the normal blood coagulation process, the excess of which caused acute coronary syndrome (ACS). In all cases, the activation of arachidonic acid by cycloxygenase was needed for the synthesis of thromboxane A_2_ (TXA_2_) but the mechanism of arachidonic acid release in platelets remains obscure. Studies were conducted to determine the role of nitric oxide (NO), if any, on the release of arachidonic acid in platelets. The cytosolic Ca^2+^ was visualized and quantitated by fluorescent spectroscopy by using QUIN-2. NO was measured by methemoglobin method. Arachidonic acid was determined by HPLC. TXA_2_ was measured as ThromboxaneB_2_ (TXB_2_) by ELISA. Treatment of platelets in platelet-rich plasma (PRP) with different aggregating agents resulted in the inhibition of nitric oxide synthase (NOS) which inhibited the production of NO synthesis and increased TXA_2_ synthesis. Furthermore, the treatment of washed PRP with different platelet aggregating agents resulted in the increase of [Ca^2+^] in nM ranges. In contrast, the pre-treatment of washed PRP with aspirin increased platelet NO level and inhibited the Ca^2+^ mobilization and TXA_2_ synthesis. These results indicated that the aggregation of platelets by different aggregating agonists was caused by the cytosolic Ca^2+^ mobilization due to the inhibition of NOS.

## INTRODUCTION

The aggregation of blood platelets by different aggregating agonists like ADP, *l*-epinephrine, thrombin or collagen is an essential physiological phenomenon ranging from the life-saving blood coagulation process to the extremes of acute coronary syndrome (ACS) [For comprehensive literature, Reference 1]. As reported earlier, the aggregation of platelets is mediated through the syntheses of prostaglandin G_2_, prostaglandin H_2_ and thromboxane A_2_ (TXA_2_) due to the catalytic action of cycloxygenase (COX) on arachidonic acid released from the platelet membrane due to the agonistic actions of the aggregating agents (2).

The excessive systemic platelet aggregation induced by the aggregating agents which may result in the thrombosis may result in ACS (1), is counter-balanced by various humoral factors that include prostacyclin (3), insulin (4), interferon-α (5), estriol (6) all of which are reported to inhibit platelet aggregation to achieve the systemic homeostasis to maintain the optimal condition for the control of excessive platelet aggregation. The inhibition of platelet aggregation by prostacyclin is mediated by the cellular increase of cyclic AMP level (7) and the inhibition of platelet aggregation by insulin (4), estriol (6) or by interferon-α (5), is reported to be mediated by the cellular synthesis of nitric oxide (NO). The inhibition of COX by acetyl salicylic acid (aspirin) resulted in the reduction of platelet aggregation without affecting cyclic AMP synthesis, however, aspirin has also been reported to inhibit platelet aggregation not only through the inhibition of COX but also through the cellular synthesis of NO (8).

It should be mentioned here that while there are many inhibitors of platelet aggregation, where almost all the agents mediate their inhibitory effect through the increase of cyclic AMP or cyclic GMP or through the increase of NO, only few agonists are currently available that can aggregate human blood platelets. Only ADP, *l*-epinephrine, collagen, thrombin, recently discovered dermcidin isoform 2 (dermcidin) which is a potent endogenous inhibitor of NO (9), and *l*-NAME (N^G^-methyl-*l*-arginine acetate ester), a well known exogenous inhibitor of NO synthase as well as only known synthetic chemical that was capable of aggregating platelets (10). Despite the fact that there are only few platelet aggregating agents currently known, no common mediator for different platelet aggregating agents is known. It is generally believed that many of these aggregating agents mediate their effect through different or even unknown pathways, and the aggregation by these aggregating agents was effected through ADP dependent activation of COX pathway (2). In other words, not all aggregating agents are of equal importance in the aggregation of human blood platelets.

Unfortunately however, the mechanism of arachidonic acid release from the platelet membrane phospholipids by all aggregating agents, essential for the initiation of the COX pathway is currently not available.

We here present herein, the effect of decreased basal NO level in platelets in the release of Ca^2+^ and its consequent effect in the release of arachidonic acid that affected the COX induced platelet aggregation.

## MATERIALS AND METHODS

### Ethical Clearance

The research project, “Involvement of nitric oxide on cytosolic calcium mobilization and arachidonic acid pathway activation during platelet aggregation with different aggregating agonists” required nominal amount of blood (5 mL) from the normal male and female volunteers between the ages of 25 to 50 years old. The INSTITUTIONAL REVIEW BOARD, HUMAN & ANIMAL RESEARCH ETHICS COMMITTEE, SINHA INSTITUTE OF MEDICAL SCIENCE AND TECHNOLOGY, Kolkata, India approved the study on the condition that followed the approved Human Ethics Protocol strictly in accordance with 1964 Helsinki declaration and no deviation in the study was allowed without the prior written permission of the board. The volunteers who participated in the study must be over ages of 25 years. No mentally retarded, pregnant women or prisoner took part in the study. All the volunteers signed an informed consent form prior to their participation in the study. It was ensured that the volunteers had no other life threatening infection. Care was taken to see that none of the volunteers were hospitalized for any condition within the last 6 months. Their complete blood picture was studied intensely and only those volunteers, who were willing to participate, were selected. Nominal amount of blood samples were drawn under the supervision of the attending physician and nurses. Seepage of blood after withdrawn, the blood was controlled by appropriate technique if any. Written consent was obtained from each of the volunteers. The committee inspected the progress and problems of the current investigation routinely.

### Chemicals & Supplies

ADP, collagen, thrombin, epinephrine, thromboxane B_2_ (TXB_2_) and TXB_2_ antibody, Quin-2/acetoxymethyl ester (AM)- a fluorescent indicator dye for Ca^2+^, EGTA, arachidonic acid, NG-methyl-*l*-arginine acetate ester (*l*-NAME) and Goat anti-rabbit immunoglobulin G-alkaline phosphatase were the products of Sigma Chemical, St Louis, MO, USA. Dermcidin isoform-2 was prepared as described before (9). Acetyl salicylic acid (aspirin) was obtained from Medica Zydus Healthcare. The insulin used in our study (Human Mixtard, Novo Nordisk India Ltd., Bangalore, India) was the product of Novo Nordisk and Eli Lily. Maxisorp plates for the enzyme linked immunosorbent assay (ELISA) were the products of Nunc, Roskilde, Denmark. Rests of the chemicals used in the study were of analytical grade. All aggregating agents and inhibitors of aggregation like aspirin were dissolved in 0.9% NaCl and neutralized to pH 7.0 at 0°C and discarded after use. 5 μM Quin-2 added from 10 mM stock in dimethyl sulfoxide (DMSO).

### Selection of volunteers

Equal number of both male and female volunteers (n=40 between the ages of 25–50 years) participated in the study. At the time of presentation none of the volunteers had any history of diabetes mellitus, or systemic hypertension or were suffering from cardiovascular disease or infection. All participants were asked to stop taking any medication including aspirin at least for 3 weeks before they were asked to participate in the study.

### Preparation of Platelet rich plasma (PRP)

Blood (5 mL) was drawn from both male and female volunteers by using 19-gauge siliconized needle and kept in plastic vials, anticoagulated by using sodium citrate as the anti-coagulant. The PRP was prepared as described before (8).

### Preparation of washed platelet suspension

PRP was centrifuged at 2000 g for 20 min at 23°C, and the platelet pellet thus obtained was washed 3 times by centrifugation after resuspending the pellet in Kreb’s buffer, pH 7.4, containing 121 mM NaCl, 3 mM KCl, 10.0 mM Glucose, 0.4 mM KH_2_PO_4_, 0.24 mM MgSO_4_, 1.73 mM CaCl_2_, 25 mM NaHCO_3_ in 250 ml distilled water, containing 1.0 mM EDTA. After the final wash the pellet was resuspended and washed 3 more times in the same buffer without EDTA containing 2.0 mM CaCl_2_.

### Aggregation of platelets

The aggregation of platelets in PRP was induced by adding 4.0 μM ADP, 5 μM epinephrine, 2 μg/ml collagen, 1 U/ml thrombin and 0.1μM dermcidin and 0.1 mM *l*-NAME and the platelet aggregation was studied by using an aggregometer (SEAC, Clot 2S, Italy) as described before (8).

### Determination of NO synthesis

NO was determined by continuously recording the spectral changes for the conversion of oxyhemoglobin to methemoglobin by the decrease of absorbance maxima at 575 nm and 630 nm in the reaction mixture containing Krebs buffer, pH 7.4, with 15 mM oxyhemoglobin in a total volume of 2.5 ml for 30 min at 37°C under N_2_ with constant stirring using a scanning Beckman spectrophotometer model DU-6 as described before (11, 21). A standard curve was constructed using pure NO in 0.9% NaCl under identical conditions as described before (11, 12). Amounts of NO in the reaction mixture were verified by using an independent chemiluminescence technique (13).

### Preparation of cell free supernatant from the disrupted platelet mass

The PRP (30 mL) was prepared from a single donor and centrifuged at 10,000g for 30 min at 0°C. The platelet mass was resuspended in 1.0ml of Kreb’s buffer pH 7.4 and disrupted by freezing and thawing in liquid N_2_. The disrupted mass was centrifuged at 30,000g, for 30 min at 0°C. The supernatant was collected and used as the source of NOS.

### Lineweaver-Burk Plot of the NOS activity of the supernatant from the disrupted platelet mass

Typically 0.2 mL of the supernatant of the disrupted platelet mass was treated with 2.0 mM CaCl_2_ in the presence or absence of 2 μM ADP, 2 μg/ml collagen, 5 μM epinephrine, 1 U/ml thrombin, 0.1 μM dermcidin and 0.1 mM *l*-NAME and different amounts of *l*-arginine (substrate of NOS) in a total volume of 1.0 mL in Kreb’s buffer pH 7.4 and incubated for 5min at 37°C. The formation of NO in the supernatant was determined by methemoglobin method as described above.

### Quin-2 labeled histochemistry using Fluorescence microscopy

Washed PRP was incubated with 5 μM Quin-2/AM in DMSO at 37°C for 30min. The cells were then centrifuged for 20 min at 350 g, as much as possible the supernatant plasma was removed, and the pellet resuspended in Kreb’s bicarbonate buffer (pH 7.4). The cells were then imaged by using a fluorescent microscope attached to a high resolution digital color camera that photographically recorded the non-weighted images of the sections. Appropriate control experiments were performed by using DMSO as used as the solvent for Quin-2/AM.

### Determination of concentration of Ca^2+^ in platelet cytosol

Washed PRP with buffer excluding calcium was loaded with 5 μM Quin-2/AM as described above. The cell suspension was then kept at room temperature in a stoppered plastic tube. Before measurements were made, aliquots of the suspension were equilibrated at 37°C for several minutes. The measurement of [Ca^2+^] from the fluorescence of intracellularly trapped Quin-2 was done by methods as described before (14-15). The cell suspension was placed in a cuvette continuously stirred using a magnetic stir bar in a specially constructed thermostatted holder in a flourimeter (Model: F-7000 FL Spectrophotometer) followed by the addition of all the aggregating agents as described before. The signal with excitation at 339 nm and emission at 500 nm was recorded on a chart recorder. For calculation of [Ca^2+^], C=k_d_ * S/(1-S) is used where C is the [Ca^2+^], K_d_ for Quin-2 is 115 nM and S is the ratio of fluorescence intensity.

### Extraction of fatty acid from platelet membrane

The platelets from washed PRP (30 mL) were disrupted by repeated freezing and thawing in liquid N_2_. The disrupted mass was centrifuged at 30,000 g for 30 min at 0°C and the pellet which contained platelet membranes was resuspended in Kreb’s buffer. The membrane suspension was incubated with 1 nM CaCl_2_ at 37°C for 10 min. The lipids were extracted by centrifuging the sample at 3000-4000 rpm to obtain cell free plasma (CFP). To the CFP, 1(N) HCl and Chloroform/Ethanol mixture was added and was mixed. The mixture was then centrifuged and the supernatant was extracted, evaporated under vacuum and stored at -70°C for High performance liquid chromatography (HPLC) analysis (17).

### HPLC Analyses

The fatty acids were separated at room temperature (26°C) using a Waters Associates (Milford MA) Model with Model 515 solvent delivery pumps, a Rheodyne injector and a model 486 tunable absorbance detector operated at 280 nm. Separation was achieved on Waters C_18_ (ID: HiQ Sil C_18_ HS) column with size 4.0 mm × 250-mm. The solvent system consisted of a mixture of water and acetic acid and acetonitrile. A sample volume of 10 μL was injected and the flow rate was maintained at 2 ml/min. All injections for quantitative measurements were made with Hamilton syringe.

### Measurement of prostaglandin synthesis in platelets

The prostaglandins synthesis was determined as TXA_2_ in its TXB_2_ form by ELISA by using TXB_2_ antibody and TXB_2_ as antigen.

### Evaluation of chelating action of EGTA on the calcium release and prostaglandin synthesis

Typically 5 mM EGTA was added to washed PRP and incubated for 30 min at 37°C. The cell suspension was further used for measurement of Ca^2+^ release and TXA_2_ formation as described above.

### Statistical Analysis

The obtained results are presented as mean ± SD, while the significance of the results was determined by using Student’s t-test. Values of *p*<0.05 were considered significant. Where appropriate, the Pearson’s correlation coefficients (r) of the results were also determined. Pearson score “*r*”, is such that -1 ≤ r ≤ +1 is acceptable where the (+) and (-) signs are designated as positive linear correlations and negative linear correlations, respectively. GraphPad Prism software (GraphPad Software, San Diego, USA) and Micro Cal origin 6.0 software were used for the statistical analyses.

## RESULTS

### Effects of different platelet aggregating agents on the basal NO level in platelets and the synthesis of TXA_2_

It has been reported before, that the aggregation of platelets induced by ADP resulted in the decrease of basal NO synthesis in platelets with concomitant increase of TXA_2_ synthesis leading to the aggregation of platelets (10) which was similar to that described in the Result section.

**Figure 1:**
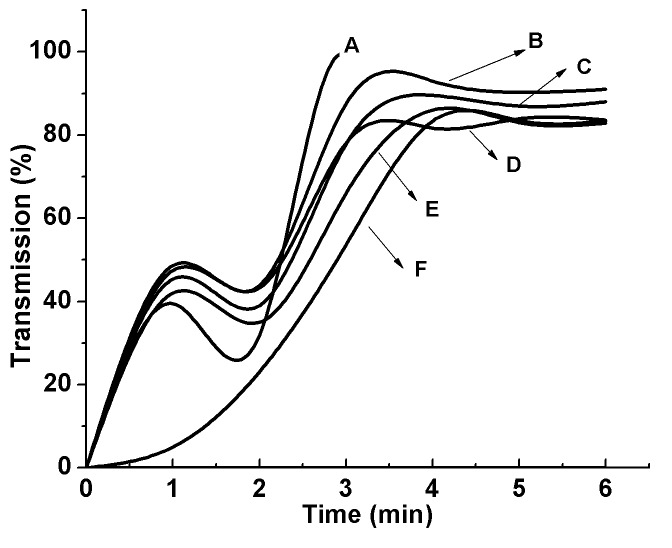
Aggregation of platelets by different aggregating agents. Different aggregating agents including ADP, epinephrine, collagen, thrombin and dermcidin and *l*-NAME was added to the PRP and the aggregation of platelets at different time intervals was studied as shown: Curve A= aggregation of PRP in case of 5 µM thrombin; Curve B= aggregation of PRP in case of 2µM ADP; Curve C= aggregation of PRP in case of 0.1 µM dermcidin; Curve D= aggregation of PRP in case of 5 µM epinephrine; Curve E= aggregation of PRP in case of 0.1 mM *l*-NAME; Curve F= aggregation of PRP in case of 2 µg/ml collagen. The upward increase of the transmission in curve A was due to the clotting of the washed PRP induced by thrombin. The figure is a typical representative of at least 10 different experiments using blood from both male and female volunteers (M=5, F=5).

**Figure 2:**
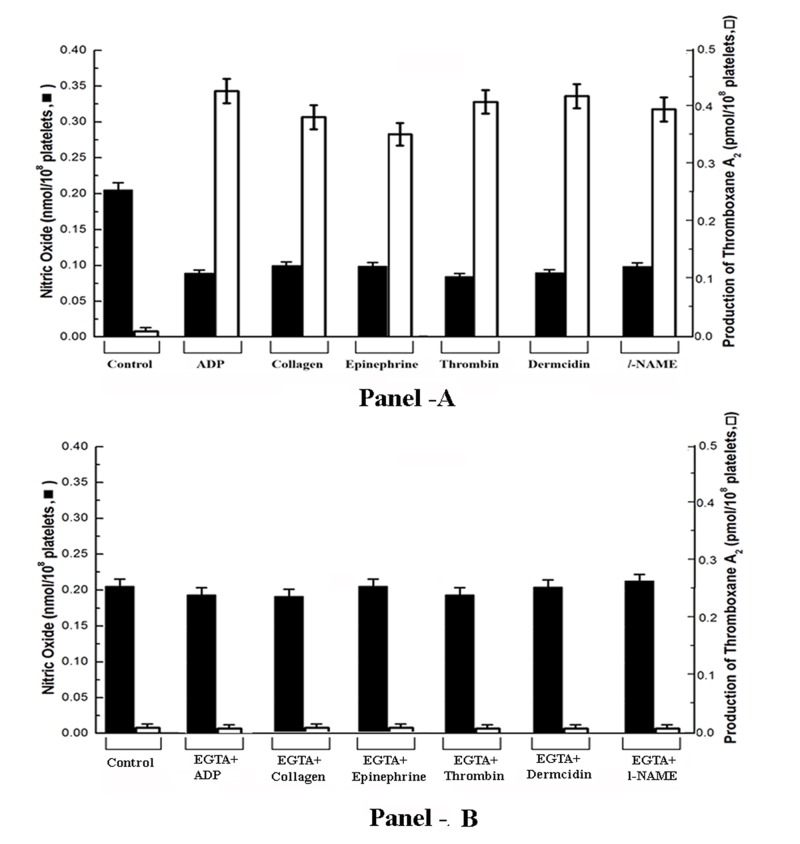
The syntheses of NO and TXA_2_ in platelets treated with different aggregating agents. Washed PRP was incubated with 4 µM ADP, 5 μM epinephrine, 2 μg/ml collagen, 1 U/ml thrombin and 0.1 μM dermcidin and 0.1 mM *l*-NAME and synthesis of NO (left Y-axis) and TXA_2_ (right Y-axis) was measured at 5 mins when the maximal platelet aggregation was achieved (Panel-A). Washed PRP was pre-treated with EGTA with further incubation with all the aggregating agents and NO and TXA_2_ was plotted (Panel-B). ‘Control’ in the figure indicates washed untreated PRP. Correlation between NO and TXA_2_ was determined by Pearson’s test and r was found to be –0.969 (*P*<0.001) indicating highly but negatively correlated. Solid square (■) indicates NO synthesis and open square (□) indicates TXA_2_ synthesis. Result shown are mean ± SD of at least 20 different experiments each in triplicate using washed PRP from 20 different subjects.

When different aggregating agents including *l*-epinephrine, collagen, thrombin as well as dermcidin were used instead of ADP, it was found that it in all cases, resulted in the aggregation of platelets within 5min as in the case of ADP (Figure 1) and the basal NO level in platelets was found to be decreased with simultaneous increase of TXA_2_ synthesis (Figure 2). It was found, as described in the Figure 2, that the increase of TXA_2_ synthesis was correlated with the reduction of NO level in platelets. The correlation determined by Pearson’s coefficient of correlation, “r” was equal to -0.969 (*P*<0.001; n=20). The negative Coefficient of correlation indicates that they (NO and TXA_2_) are highly but negatively correlated.

Furthermore, the use of *l*-NAME, a well known inhibitor of NOS that is reported to decrease the basal NO level in platelets (10), was also found to aggregate platelets (Figure 1, Curve E) with simultaneous decrease of the basal NO level that resulted in the increased TXA_2_ synthesis (Figure 2) as in the cases of “physiologic” platelet aggregating agents as described above. These as a whole demonstrated that there exists a direct relationship between aggregation of platelets and the reduction of the basal NO level which further led to the increased TXA_2_ production in the platelets leading to aggregation of platelets.

### Line-weaver Burk plot of the nitric oxide synthase (NOS) in the presence or absence of different aggregating agonists

Line weaver Burk plot of the NOS present in the cytosolic fraction of the disrupted platelet mass in the presence and absence of different aggregating agents including 4 µM ADP, 2 µg/ml collagen, 5 µM epinephrine, 1 U/ml thrombin, 0.1 µM dermcidin and 0.1 mM *l*-NAME was constructed. It was found in the presence of these aggregating agents resulted in the inhibition of the NOS as described in Table 1. The Michaelis constant (Km) of the NOS (control) was 5.26 µM arginine with maximum velocity (Vmax) of 6.66 nmol NO formed/h/mg protein. The addition of the aggregating agents in the reaction mixture as in the case of thrombin reduced the Vmax with simultaneous increase of Km indicating that the rate of NO synthesis in the reaction mixture in the presence of different aggregating agents reduced by nearly 70% (*P*<0.01) *in vitro* (Table 1).

**Table 1:** Line-weaver Burk plot of the inhibition of NOS induced by different aggregating agents in the cell free supernatant from the disrupted platelet mass

Addition to PRP	[Ca^2+^] (nM)

Collagen (2 μg/ml)	128 ± 12.50
Epinephrine (5 μM)	134 ± 10.50
Thrombin (1 units/ml)	147 ± 9.50
Dermcidin (0.1 μM)	123 ± 5.50
*l*-NAME (0.1 mM)	124 ± 6.70

The cell free supernatant from the disrupted platelets was prepared as described in Methods and Materials. Line–weaver Burk plot was constructed by adding different amounts of *l*-arginine to the reaction mixture in the presence and absence of different aggregating agents as shown. The Vmax when “None” was added to the disrupted platelet mass and when all the aggregating agents were added to the disrupted platelet mass was found to be statistically significant (*P*<0.01). Each point represents mean of 5 different experiments each in triplicate.

### Intracellular mobilization of Ca^2+^ in washed PRP treated with different aggregating agents

**Figure 3:**
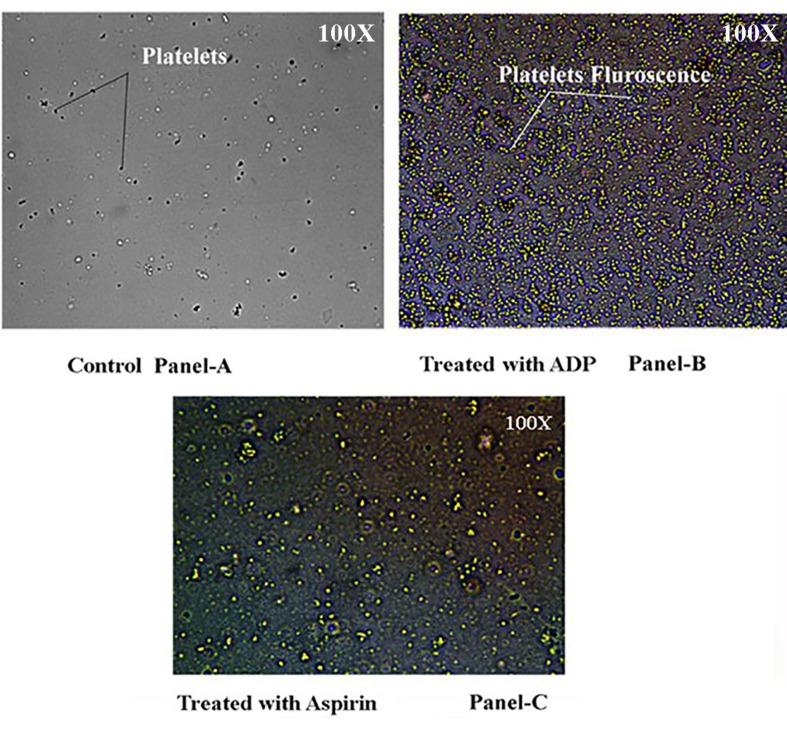
Visualization of the cytosolic Ca^2+^ in platelets treated with Quin-2/AM by fluorescence microscopy in the presence of ADP. Washed platelets were loaded with 5 μM QUIN-2/AM with ssubsequent addition of 4.0 µM ADP or with and 80 µM aspirin together when required to the reaction mixture and are mounted on a glass slide to be seen in a fluorescent microscope. Panel-3A shows the presence of non-aggregated platelets (black arrows) in the presence of DMSO (Vehicle for QUIN-2/AM) only. Panel-3B shows the platelets treated with QUIN-2 and ADP. The presence of aggregated platelets (shown with arrows) with yellow color fluorescence is seen due to the interaction of Ca^2+^ with QUIN-2. Panel-3C shows the QUIN-2 labeled platelets treated with both aspirin and ADP. The decrease in intensity of the yellow fluorescence is seen due to the effect of aspirin on blocking the interaction of Ca^2+^ with Quin-2. The magnification used in each case shown in the top right corner of the figure. The significance of the fluorescence was determined by the amount of Ca^2+^ released from the platelet dense granule as given in Figure 4. The figures shown are typical representative of six different experiments using 6 different human washed PRP samples.

To determine the effect of ADP on the mobilization of Ca^2+^ in the platelet cytosol, washed PRP was “loaded” with QUIN-2/AM and subsequently treated with 4 μM ADP without the presence of external Ca^2+^, the fluorescence of Quin-2/AM due to the interaction with free Ca^2+^ was demonstrated (Figure 1, Panel-B) compared to control (Figure 3, Panel-A).

**Figure 4:**
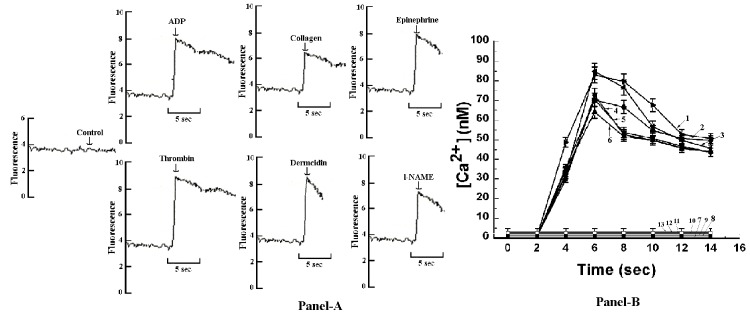
Assessment of cytosolic Ca^2+^ mobilization in Quin-2 loaded platelets in the presence of all the aggregating agents by fluorescence spectrophotometric assay. Washed platelets were treated with 4 µM ADP and quantization of [Ca^2+^] was performed using fluorescence spectrophotometer. Panel-4A shows fluorescence recordings in response to the addition of all the aggregating agents to the Quin-2 loaded platelet suspension. The increase of cytosolic Ca^2+^ was determined by fluorescence spectrophotometer by determining fluorescence of free Ca^2+^ due to interaction of Ca^2+^ with Quin-2/AM. Panel-4B shows the increase of intracellular [Ca^2+^] treated with all the aggregating agents at different time intervals in the absence of external calcium. Line 1 indicates 1 U/ml thrombin, Line 2 indicates 0.1 µM dermcidin, Line 3 indicates 4 µM ADP, Line 4 indicates 5 µM epinephrine, Line 5 indicates 0.1 mM *l*-NAME, Line 6 indicates 2 µg/ml collagen, Line 7 indicates control (washed untreated PRP) and Line 8 -13 indicates [Ca^2+^] in case of washed PRP pre-treated with 5 mM EGTA along with addition of all the aggregating agents. The peak height of [Ca^2+^] in untreated platelets and in platelets treated with all the aggregating agents was found to be statistically significant where *P*<0.01 determined by Student´s t-test. Addition of the aggregating agents was made by syringe directly into the cuvette while the portions of platelet suspension were continuously stirred. Results shown are mean ± S.D. of atleast 15 other experiments each in triplicate using blood samples from 15 different volunteers (M=10, F=5).

In a related study, fluorescence record (Figure 4, Panel-A) and amount of Ca^2+^ released (Figure 4, Panel-B) from the cells stimulated with different aggregating agents was measured in a fluorimeter. It was found that at 7seconds of addition of different aggregating agents, [Ca^2+^] reached its peak height of 72.0 ± 3.6 nM in case of ADP, 63.4 ± 3.17 nM in case of collagen, 69.8 ± 3.49 nM in case of epinephrine, 84.1 ± 4.20 nM in case of thrombin, 82.3 ± 4.11 nM in case of dermcidin and 69.5 ± 3.47 nM in case of *l*-NAME. In all cases, the increase in peak height of [Ca^2+^] in untreated platelets and in platelets treated with the aggregating agents was found to be statistically significant where *P*<0.01.

### Role of decreased NO level on the release of arachidonic acid from the platelet membrane phospholipid needed for the synthesis of TXA_2_

**Figure 5:**
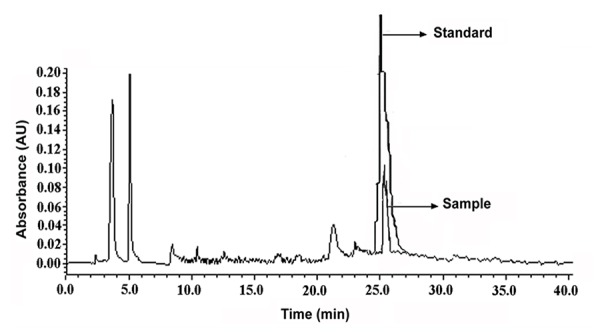
HPLC assessment of arachidonic acid release from isolated platelet membrane preparation treated with exogenous Ca^2+^. Platelet membrane preparation (2 mg) was treated with 1nM extraneous Ca^2+^ (CaCl_2_). The fatty acids were extracted and chromatographed in HPLC. For comparison, pure sample of arachidonic acid (Sigma) was used as standard as shown. “Sample” in the figure means isolated platelet membrane preparations after exogenous calcium incubation and “Standard” means characteristic HPLC peak for pure arachidonic acid. The sample absorbance was found to be statistically significant where *P*<0.01 determined by Student t-test. The results shown here is a typical representative of 7 different experiments using washed PRP from 7 different volunteers.

It was thought that the reduction of platelet NO level by different aggregating agents resulted in the intracellular mobilization of Ca^2+^ which remains sequestered in the dense granules in the platelet cytosol might activate intrinsic phospholipase A_2_ present in the platelet membrane leading to the release of arachidonic acid. To verify this possibility, when isolated platelet membrane was incubated with 1 nM Ca^2+^, the release of 8.5 nmole of arachidonic acid/mg was found to occur as determined by HPLC (Peak retention time was 25.66 mins, Figure 5).

### Effect of calcium chelation on NO, release of Ca^2+^ from the dense granules and the TXA_2_ formation

As described above, the decrease of basal NO level was responsible for the release of intracellular Ca^2+^ from the platelet dense granule which affected the arachidonic acid release and resulted in TXA_2_ formation leading to subsequent aggregation of platelets. To determine the effect of a well-known calcium chelating agent like EGTA on the above process, washed PRP was pre-treated with 5 mM EGTA with further addition of the different aggregating agents as described before. No fluorescence peak ([Ca^2+^]) could be seen in fluorescence spectrophotometer in case of all aggregating agents (Figure 4, Line 8-13). On the other hand, presence of basal NO level in platelets was observed with simultaneous inhibition of TXA_2_ formation (Figure 2, Panel-B).

### Effect of increase of platelet NO level by aspirin on the aggregation of platelets by different aggregating agents

The treatment of washed PRP with 80μM aspirin, 200 μU/ml insulin or with 0.8 nM NO in 0.9% NaCl as described in the Materials and Methods rendered the platelets resistant to the aggregating effect of all aggregating agent as described above with decreased platelet TXA_2_ synthesis (Table 2).

**Table 2:** The effect of treatment of washed PRP with aspirin in the syntheses of NO, TXA_2_ and % of aggregation

Addition in PRP	NO formation (nmol/10^8^ platelets)	TXA_2_ formation (pmol/10^8^ platelets)	Transmission (%)

None	0.250 ± 0.004	0.010 ± 0.005	0
Aspirin (80 μM)	0.340 ± 0.005	0.010 ± 0.005	0
ADP (4 μM)	0.352 ± 0.010	0.155 ± 0.007	9.8 ± 0.490
Collagen(2 μg/ml)	0.364 ± 0.010	0.154 ± 0.007	7.7 ± 0.385
Epinephrine (5 μM)	0.354 ± 0.020	0.152 ± 0.007	8.4 ± 0.420
Thrombin (1 units/ml)	0.353 ± 0.020	0.148 ± 0.006	12.2 ± 0.610
Dermcidin (0.1 μM)	0.359 ± 0.020	0.143 ± 0.007	8.7 ± 0.435
*l*-NAME (0.1 mM)	0.400 ± 0.020	0.151 ± 0.004	7.9 ± 0.395

Washed PRP was treated with 80 μM aspirin and the syntheses of NO, TXA_2_ were determined after the initiation of aggregation by different aggregating agents. Aggregation of platelets at 5mins is described as % of transmission. The increase of NO synthesis in platelets pre-treated with aspirin with subsequent addition of different aggregating agents was found to be related to the decrease of TXA_2_ formation and in the reduction of transmission of light as determined by Pearson’s correlation coefficient, “r” which was equal to -0.985 (*P*<0.001, n=20). The figure shown here is a typical representative of at least 10 more identical experiments.

When the platelets were pre-incubated with 200 μU/ml insulin instead of aspirin and subsequently 4 μM ADP added, the NO level increased from the basal 0.08 ± 0.001nmol to 0.367 ± 0.02 nmol/10^8^ platelets (*P*<0.001, n=10) which resulted in the inhibition of aggregation of platelets by nearly 60% with simultaneous reduction of TXA_2_ from 0.35 ± 0.02 pmol in the presence of 4.0 μM ADP to 0.158 ± 0.007 pmol/10^8^ platelets (Pearson’s correlation coefficient, “r” which was equal to -0.972 (*P*<0.001; n=20).

### The effects of increased platelet NO level on the ADP induced release of arachidonic acid, Ca^2+^ mobilization in platelets

When the washed PRP was incubated with either 80 µM aspirin or 200 µU/ml insulin or 0.8 nM NO followed by the addition of 4 µM ADP, no release of arachidonic acid from the platelet membrane was observed in HPLC and as seen without any change in transmission in fluorimeter. The treatment of Quin-2 loaded washed PRP with 80 µM aspirin demonstrated the presence of lesser amount of fluorescence similar to control (washed PRP only) as shown in Figure 3, Panel-C.

### Role of l-NAME in the release of arachidonic acid and Ca^2+^ mobilization in washed PRP

It could be argued that the effect of ADP on TXA_2_ synthesis is reported to be due to the ADP related activation of platelets (18). However, the use of *l*-NAME, an inhibitor of NOS and also an inducer of TXA_2_ synthesis (Figure 2), produced similar effects on arachidonic acid release (7.2 nmole of arachidonic acid/mg) and Ca^2+^ mobilization in washed PRP ([Ca^2+^]= 124 ± 6.70 nM) independent of ADP related interaction.

## DISCUSSION

These results suggest that the basal NO level in platelets could be important both in the aggregation and in the inhibition of aggregation of platelets induced by all currently known platelet aggregating agents.

It has been reported before, that the platelet aggregation induced by different aggregating agonists was actually due to the effect of ADP induced platelet aggregation and as a result of the “activation” of platelet COX led to the synthesis of TXA_2_ for the aggregation of platelets (2). However, it must be mentioned here, that for the process of aggregation of platelets by ADP, the availability of arachidonic acid, the only substrate for COX is essential. No acceptable explanation is currently available for the arachidonic acid release from the platelet membrane phospholipids which could only be achieved by the activation of phospholipase A_2_ (2). In other words, ADP itself cannot release platelet membrane phospholipids for the activation of COX as the release of arachidonic acid is not an ADP-specific event.

We have reported before that ADP was both an activator of COX as well as an inhibitor of cytosolic NOS (10). In this context, the claim that suggested that it was the ADP itself that was the “protagonist” in the platelet aggregation induced by different aggregating agents including *l* epinephrine, collagen or thrombin. Our results nevertheless showed that all currently known platelet aggregating agents as described above including dermcidin isoform 2 or even *l*-NAME were capable of reducing the basal NO level in platelets leading to the platelet aggregation to follow (Figure 1 and Figure 2). The reduction of NO level resulted in the Ca^2+^ mobilization in platelet cytosol leading to the synthesis of TXA_2_ through the liberation of arachidonic acid due to the activation of intrinsic membrane phospholipase A_2_. And, as such, the above platelet aggregating agents including ADP were equivalent in their effect in platelet aggregation through the mobilization of Ca^2+^ leading to TXA_2_ synthesis through the COX pathway.

The results also demonstrated that the decrease of basal NO level in platelets resulted in the appearance of Ca^2+^ in the platelet cytosol as visualized in fluorescence microscopy using Quin-2 labeled platelets (Figure 3). In a similar study, when cytosolic Ca^2+^ mobilization in Quin-2 loaded platelets in the presence of all the aggregating agents were assessed, it was found that in case of ADP, 72 nM of [Ca^2+^] was released at 7seconds (Figure 4, Panel-B, Line 3) with simultaneous increase in cell fluorescence (Figure 4, Panel-A). It should be noted here, that the treatment of platelets with EGTA followed by the addition of all the aggregating agents didn´t affected the basal NO level in platelets (Figure 2), nullified the release of platelet Ca^2+^ from the dense granules (Figure 4, Panel-B, Line-8) and consequently inhibition of TXA_2_ (Figure 2) formation in platelets was observed.

Furthermore, the availability of Ca^2+^ in the cytosol resulted in the synthesis of TXA_2_ due to availability of arachidonic acid (Figure 5). That arachidonic acid could be released from the platelet membrane phospholipid directly demonstrated by treating platelet membrane with Ca^2+^ as evidenced by HPLC (Figure 5). In this context, it can be mentioned here that even the addition of ADP along with all other aggregating agents to the platelet membrane preparation (in the absence of Ca^2+^ in the incubation mixture) did not release arachidonic acid suggesting that these agents by itself was not the cause for the release of arachidonic acid from platelet membrane phospholipids. Indeed all known aggregating agents including ADP were following similar metabolic pathway for the release of arachidonic acid irrespective of their receptor activity, indicating that the release of the platelet membrane arachidonic acid is a “post receptor interaction” phenomenon leading to the increase of the cytosolic Ca^2+^ through the inhibition of platelet cytosolic NOS.

**Figure 6:**
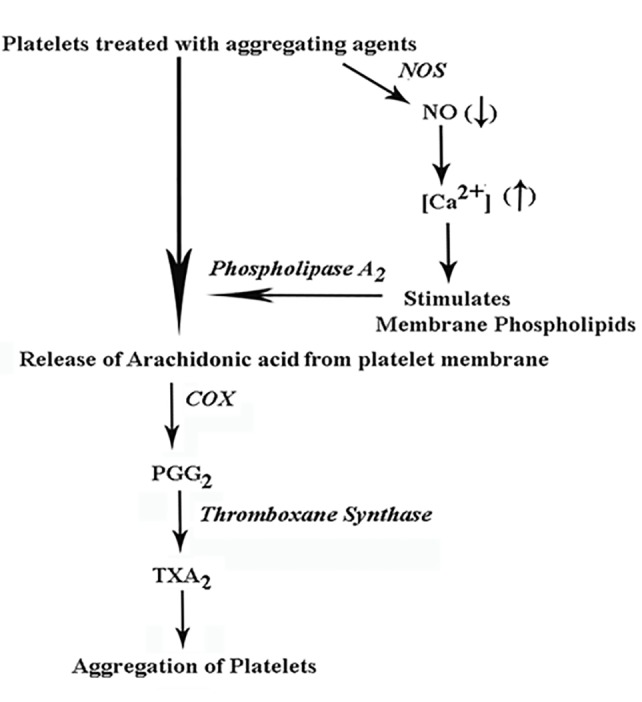
Schematic representation of the mechanistic action of the reduction of NO level in the release of arachidonic acid in platelets treated with different aggregating agonists. The figure demonstrates the role of the reduction of the NO due to the inhibition of cyosolic NOS that led to the release of Ca^2+^ from the dense granules. The released Ca^2+^ subsequently involved in the activation of phospholipase A_2_ catalyzed arachidonic acid release from the platelet membrane. NO= nitric oxide; NOS= nitric oxide synthase; COX= cycloxygenase; PGG_2_= prostaglandin G_2_; TXA_2_= thromboxane A_2_.

In each case of the aggregating agonists, irrespective of the nature of receptors involved, the aggregation of platelets was found to be related to the decrease in the rate of the NOS (Vmax) with corresponding changes in the optimum substrate concentration (Km) for *l*-arginine, the substrate for NOS (Table 1). These results suggested that the inhibition of the platelet cytosolic NOS by different aggregating agents, natural or synthetic resulted in the inhibition of the basal NO level leading to the release of arachidonic acid from the platelet membrane phospholipids due to the activation of intrinsic phospholipase A_2_ by Ca^2+^ (Figure 6).

Although it has been repeatedly claimed that ADP was capable of activating platelet membrane COX, the availability of arachidonic acid in platelets was adequate to produce TXA_2_ (Figure-2) even in the absence of the added ADP to the platelet preparation (Figure 5) which led to the reduction of platelet NO level leading to the synthesis of TXA_2_ and to the aggregation of platelets (Figure 1). The increase of basal level of NO either by aspirin (12) or by insulin (4) or by treating washed PRP with 0.9 nM NO in 0.9%NaCl all resulted in the inhibition of platelet aggregation, inhibition of TXA_2_ synthesis with no appearance of Ca^2+^ in the platelet cytosol (Table 2). These results also indicated that the effect of aspirin as the useful therapeutic agent which not only inhibited COX but also increased NO synthesis that resulted in the inhibition of aggregation of platelets.

Finally it should be mentioned here that NO has been reported to act as the biologic “messenger molecule” before (19) and the cellular Ca^2+^ has been reported to be a biologic messenger in various cellular events (20). Our results as reported above suggested that the NO effect as the antecedent biologic messenger for the mobilization of platelet cytosolic Ca^2+^ and for the activation of COX for the aggregation of platelets to follow. Conversely increase of NO level in platelets associated with the inhibition of platelet aggregation which resulted in the inhibition of both TXA_2_ synthesis and in the appearance of Ca^2+^ in the platelet cytosol.

In conclusion, it could be summarized that the inhibition of NO synthesis by different aggregating agents resulted in the increased intracellular mobilization of Ca^2+^. The Ca^2+^ thus liberated from the dense granule of the platelet cytoplasm in turn activated Phospholipase A_2_ releasing arachidonic acid from the platelet membrane phospholipids and thus affected the aggregation of platelets through the COX induced prostaglandin synthesis.
